# Longitudinal Study on Clinical Predictors for Allergic Bronchopulmonary Aspergillosis in Children and Young People with Cystic Fibrosis Highlights the Impact of Infection with *Aspergillus* and *Pseudomonas* and Ivacaftor Treatment

**DOI:** 10.3390/jof11020116

**Published:** 2025-02-04

**Authors:** Emily L. D. Chesshyre, Beth Enderby, Angela C. Shore, Fiona C. Warren, Adilia Warris

**Affiliations:** 1 MRC Centre for Medical Mycology, Department of Biosciences, Faculty of Health and Life Sciences, University of Exeter, Exeter EX4 4QD, UK; a.warris@exeter.ac.uk; 2 Department of Paediatrics, Royal Devon University Healthcare NHS Foundation Trust, Exeter EX2 5DW, UK; 3 Faculty of Health and Life Sciences, University of Exeter, Exeter EX1 2LU, UK; a.c.shore@exeter.ac.uk (A.C.S.); f.c.warren@exeter.ac.uk (F.C.W.); 4 NIHR Exeter Clinical Research Facility, Royal Devon University Healthcare NHS Foundation Trust, Exeter EX2 5DW, UK; 5 Department of Paediatric Infectious Diseases, Great Ormond Street Hospital, London WC1N 3JH, UK

**Keywords:** ABPA, *Aspergillus*, cystic fibrosis, paediatrics, risk factors

## Abstract

Allergic bronchopulmonary aspergillosis (ABPA) is a well-known complication in children and young people with cystic fibrosis (CF) and without treatment causes structural lung damage. We performed a longitudinal observational study to identify clinical risk factors for ABPA in a cohort of children and young people with CF aged 8 to 17 years at baseline. Anonymised annual review UK CF Registry data from 2009 to 2019 for patients aged 8–17 years in 2009 were collected, with lung transplant recipients excluded. Baseline characteristics are presented for the whole group and cross-sectional comparisons made according to the presence of ABPA or not in 2009. Longitudinal analysis from 2009 to 2019 was completed on the group without ABPA in 2009 to identify predictors for the subsequent development of ABPA using a complementary log–log regression model. In 2009, there were 1612 patients, of which 1420 were ABPA-negative and 192 ABPA-positive. *Aspergillus* colonisation (*p* = 0.01) and IV antibiotic use (*p* < 0.0001) were associated with having ABPA in 2009. Longitudinal analysis of the group without ABPA in 2009 identified male gender, younger age, lower lung function, *Pseudomonas aeruginosa* infection, and *Aspergillus* colonisation to be significantly associated with the development of ABPA (*p* < 0.0001). Ivacaftor was significantly associated with reduced ABPA (OR 0.46, *p* = 0.01) but not lumacaftor/ivacaftor (OR 0.64, *p* = 0.28). Chronic oral macrolide use was significantly associated with increased risk of development of ABPA (OR 1.30, *p* < 0.0001). This study shows that lower lung function, *Aspergillus* colonisation, and *Pseudomonas aeruginosa* infection in children with CF were associated with the development of ABPA, highlighting the need for enhanced surveillance in these patients. This is the first study to show a protective association of ivacaftor and ABPA.

## 1. Introduction

Cystic fibrosis (CF) is caused by mutations in the CF transmembrane conductance regular (***CFTR***) gene and is characterised by recurrent respiratory bacterial and fungal infections. Allergic bronchopulmonary aspergillosis (ABPA) caused by an exaggerated type 2 hypersensitivity response to *Aspergillus* in the airways is a severe complication of CF that without treatment causes structural lung disease [1] and poor outcomes [2,3,4]. ABPA is diagnosed from a composite of characteristic laboratory (serological markers of ***Aspergillus*** infection), radiographic, and clinical features [1]. While the prevalence of ABPA reduced in the UK since 2015, in 2022, it still affected 5% of all UK CF patients [5]. Understanding the risk factors for ABPA may enable earlier identification and treatment to improve outcomes and reduce the development of structural lung damage including bronchiectasis.

The majority of studies that have evaluated risk factors for ABPA in CF have been cross-sectional, which can overestimate the strength of associations. In addition, these studies were published before 2011, well before the introduction of CFTR modulator therapies. A large cross-sectional observational European CF Patient Registry (ECFPR) study 25 years ago including 14,210 CF patients reported a 2% ABPA prevalence (lower than other studies at the time) and identified the following as risk factors for ABPA: female gender, adolescent age, lower lung function, asthma, and infection with *Pseudomonas aeruginosa* [6]. Another ECFPR study from 24 years ago identified the following risk factors for ABPA from cross-sectional analysis: homozygous F508del, lower lung function, *Aspergillus* colonisation, and poor nutritional status [3]. A Belgian case–control CF registry study identified an association between ABPA and lower lung function and previous Dornase alfa and inhaled steroid treatment (n = 73) [7]. Other small single-centre observational studies have shown an association between ABPA and low BMI (n = 85) [8], *Stenotrophomonas maltophilia* (n = 160) [9], and IV antibiotics (n = 150) [10]. The lack of recent longitudinal data and the introduction of CFTR modulator therapy makes evaluation for risk factors for ABPA a key research priority in 2024 [11].

Ivacaftor (Kalydeco^®^) was the first CFTR modulator therapy approved in Europe in July 2012, for people with CF aged ≥6 years of age with G551D mutation, the most common gating mutation. It is a potentiator that binds to the defective CFTR protein at the cell surface and opens the chloride channel allowing chloride to flow through. Its clinical benefits for eligible patients have been shown in clinical trials [12,13,14,15] and real-world studies [16,17]. Combination CFTR modulator therapy was subsequently developed where a corrector (such as lumacaftor, tezacaftor, or elexacaftor) was added to ivacaftor, facilitating the maturation and intracellular trafficking of the CFTR protein and increasing the quantity of CFTR proteins at the cell surface, allowing many more patients to benefit from therapy. Lumacaftor/ivacaftor (Orkambi^®^) and tezacaftor/ivacaftor (Symdeko^®^) were approved in Europe for people with CF ≥ 12 years of age in 2015 and 2018, respectively, for patients with homozygous F508del and other specified mutations. Elexacaftor/tezacaftor/ivacaftor (ETI) (Kaftrio^®^/Tricafta^®^) was approved in Europe for people with CF ≥ 12 years of age in 2020 for patients heterozygous F508del or at least one copy of other specified mutations. ETI is now widely available across Europe and North America, having shown dramatic improvements in lung function and quality of life in CF in clinical trials [13,14,15,18] and now real-world studies [19,20]. With this change in clinical practice, it is, therefore, of vital importance to understand whether CFTR modulator therapies have influenced the risk of ABPA, as the clinical trials and real-world data studies to date have not yet reported on this.

The aim of this study is to evaluate the risk and protective factors for ABPA in children and young people with CF by using data from the UK CF Registry. The objectives are to identify the clinical characteristics and treatments positively and negatively associated with the subsequent development of ABPA. The identification of children with CF, needing enhanced surveillance for ABPA based on their risk profile, is of value for facilitating early detection and treatment of ABPA. Of particular interest is the impact of the early CFTR modulator therapies on the development of ABPA.

## 2. Methods

### 2.1. Study Population and Design

This is a follow-on study from our previous study on the long-term outcomes of *Aspergillus* and ABPA in children and young people with CF, which was an 11-year longitudinal observational cohort study of 1675 children with CF that showed a significant association between ABPA and increased longitudinal decline in lung function and body mass index (BMI) [4]. Anonymised annual review data were provided by the UK CF Registry (UKCFR) for children and young people who were aged 8–17 years in 2009, with annual review data provided for all years from 2009 to 2019 on age, gender, ethnicity, *CFTR* genotype, BMI percentile, lung function, positive respiratory samples (*Aspergillus* spp., *Pseudomonas aeruginosa* and other bacteria), ABPA, treatments (antibiotics and CFTR modulator therapies), and outcomes (lung transplant, death). (see Supplementary Materials for full list of variables provided). The UKCFR is a research-ethics-committee-approved research database which hold data in a secure centralised database from people with CF in the UK on clinical and demographic characteristics upon informed consent. The starting year for this follow-on study was chosen as 2009 as it was only from 2009 when annual review data were available for >80% of UK CF patients registered. Moreover, 2019 was chosen as the final year of this follow-on study prior to the COVID-19 pandemic, when 96% of UK CF patients had annual review data recorded in the CF Registry. Patients were included at the start of the study if they were recorded in the UK CF registry (UKCFR) from 1 January 2009 to 31 December 2010 and aged between 8 and 17 years. They were excluded if they did not survive after 31 December 2010 due to the lack of follow-up data or had a lung transplant prior to 2019 due to different clinical phenotypes and risk profiles (n = 62). Annual review data were provided on these patients up until 31 December 2019. This study was approved by the UKCFR Steering Committee (Huntingdon Research Ethics Committee [07/Q0104/2]).

Characteristics at baseline in 2009 are described in all patients, and those with and without ABPA in 2009 were compared. The definition of ABPA used by the UKCFR is that of the CF Foundation Consensus Criteria (1). Longitudinal analysis was then completed on those who were ABPA negative in 2009 to determine the association of predictive factors for subsequent ABPA, including age, gender, and genotype in 2009, and in the year prior to the development of ABPA: lung function measured as ppFEV_1_ (percentage predicted forced expiratory volume in 1 s), percentile BMI, *Aspergillus* spp. colonisation (≥1 positive respiratory culture for *Aspergillus* over 12 months), and *Pseudomonas aeruginosa* infection (≥1 positive respiratory culture for *P. aeruginosa* over 12 months), and treatments (pancreatic enzyme supplements, IV antibiotic days, chronic oral macrolides, ivacaftor, and lumacaftor/ivacaftor).

### 2.2. Statistical Analysis

#### 2.2.1. Baseline Characteristics: Cross-Sectional Analysis

Descriptive characteristics in 2009 are presented for all patients in the cohort and stratified according to whether they had ABPA or not in 2009. Continuous variables (age, lung function measured as ppFEV_1_, and percentile BMI) are summarised as mean and standard deviation and compared using the two-sided *t*-test. Categorical variables (gender, genotype, oxygen therapy, and non-invasive ventilation) are summarised as frequency and percentage and compared using the chi-squared test. A cross-sectional analysis was performed to identify characteristics associated with ABPA in 2009, using a multivariable logistic regression model with the following predictor variables also measured in 2009: age, gender, genotype, lung function as ppFEV_1_, percentile BMI, *Aspergillus* spp. colonisation (≥1 positive respiratory culture for *Aspergillus* over 12 months), *P. aeruginosa* infection (≥1 positive respiratory culture for *P. aeruginosa* over 12 months), and treatments (IV antibiotic use, chronic macrolide use, pancreatic enzyme supplementation). Odds ratios with 95% confidence intervals are presented.

#### 2.2.2. Longitudinal Analysis

Longitudinal analysis was completed on the group who were ABPA negative in 2009 (n = 1420) with a complementary log–log regression model to determine the variables associated with subsequent development of ABPA. The baseline predictors included in the model were age, gender, and genotype. The predictors from the year preceding the development of ABPA (i.e., predictors in year 2009 and ABPA outcome in 2010 throughout the cohort until predictors in year 2018 and ABPA outcome in 2019) included in the model were lung function measured as ppFEV_1_, percentile BMI, *Aspergillus* spp. colonisation (≥1 positive respiratory culture for *Aspergillus* over 12 months), *P. aeruginosa* infection (≥1 positive respiratory culture for *P. aeruginosa* over 12 months), and treatments (pancreatic enzyme supplements, IV antibiotic days, ivacaftor, and ivacaftor/lumacaftor). Odds ratios with 95% confidence intervals are presented. Missing data in this study were ≤15.3% for all variables, with all variables ≤ 10.0% missing other than lung function, BMI, and ABPA. All variables included in the model were binary variables, other than age (continuous), *CFTR* genotype (categorical), ppFEV_1_ (continuous), and percentile BMI (continuous).

Statistical analysis was performed using Stata v. 18. *p*-values < 0.05 were deemed statistically significant.

## 3. Results

### 3.1. Baseline Characteristics

A total of 1612 patients were included with a mean age of 11.9 years, and 51.4% were male. Moreover, 55.9% were homozygous for F508del, 37.0% heterozygous for F508del, and 7.1% had other genotypes (Table 1). The patients who had ABPA in 2009 were significantly older than those without (mean age 12.4 years versus 11.9 years, *p* = 0.008). There were no significant differences between gender or genotype between those who had ABPA in 2009 and those who did not. Markers indicating severity of respiratory disease were significantly increased in those who had ABPA in 2009 compared to those who did not: lung function (ppFEV_1_ 76.0% versus 80.9%, *p* = 0.0003), oxygen therapy (7.9% versus 2.3%, *p* < 0.0001) and non-invasive ventilation (6.2% versus 1.3%, *p* < 0.0001). Percentile BMI was significantly increased in those who had ABPA (53.6 versus 48.8, *p* = 0.04) (Table 1).

The results of the baseline cross-sectional analysis with a multivariable logistic regression model to determine the predictive characteristics associated with ABPA in 2009 are displayed in Table 2. No significant association was shown with lung function, *Pseudomonas aeruginosa* or chronic oral macrolides or pancreatic enzyme supplementation, and ABPA. A significant association was shown with ABPA and percentile BMI (OR 1.01, *p* = 0.004), *Aspergillus* colonisation (OR 2.0, *p* = 0.01), and IV antibiotic use (OR 3.59, *p* < 0.0001).

### 3.2. Longitudinal Analysis

Of the 1420 patients who were ABPA negative in 2009, 348 patients went on to develop ABPA, and 5.8% died prior to 2019. From 2013 to 2018, 165 patients were started on CFTR modulator therapies (77 on ivacaftor, 88 on lumacaftor/ivacaftor, and 1 on tezacaftor/ivacaftor) (see Supplementary Materials, Table S1).

The results of the longitudinal analysis are displayed in Table 3 and Table 4. Male gender at baseline (OR 1.45 (1.28–1.65), *p* < 0.0001), *Aspergillus* colonisation (OR 1.47 (1.28–1.69), *p* < 0.0001), and *P. aeruginosa* (OR 1.31 (1.15–1.49), *p* < 0.0001) in the year prior to the year of development of ABPA were significantly associated with the subsequent development of ABPA. Reduced lung function (OR 0.98 (0.98–0.99), *p* < 0.0001) and younger age (OR 0.89 (0.87–0.91), *p* < 0.0001) were also significantly associated with the development of ABPA. There were no significant associations between ABPA and *CFTR* genotype or BMI (Table 3).

Ivacaftor was significantly associated with a reduced development of ABPA (OR 0.46 (0.26–0.80), *p* = 0.01) but not lumacaftor/ivacaftor (OR 0.64 (0.29–1.44), *p* = 0.28). Chronic macrolide use was associated with an increased risk to develop ABPA (OR 1.30 (1.15–1.48), *p* < 0.0001) but not IV antibiotic use (OR 1.0, *p* = 0.24) or pancreatic enzyme supplementation (OR 0.86, *p* = 0.22) (Table 4).

## 4. Discussion

This large real-world longitudinal cohort study shows that in children with CF, lower lung function, *Aspergillus* colonisation, and *P. aeruginosa* infection were associated with the development of ABPA. Furthermore, it is the first real-world study to show a protective association of ivacaftor on the subsequent development to ABPA.

The positive association found in our study between *Aspergillus* colonisation and subsequent ABPA is interesting and important. While *Aspergillus* needs to be present for the type 2 inflammatory response to occur that leads to ABPA, *Aspergillus* colonisation is not one of the major international consensus diagnostic criteria for ABPA, due to a cited lack of evidence of specificity, particularly in asthma [1,21,22]. In our previous UKCFR study, we demonstrated that, in children with CF and *Aspergillus* colonisation, 30.4% had ABPA, and in those who had ABPA 30.8% had *Aspergillus* colonisation [4]. In an older ECFPR study including 12,447 children and adolescents with CF, in patients with *Aspergillus* colonisation, there was a 19.6% prevalence of ABPA, compared to an overall prevalence of ABPA of 8.1% [3]. In view of these findings, enhanced surveillance for ABPA in children and young people with CF colonised with *Aspergillus* spp. may be beneficial for early intervention. Enhanced clinical and radiological monitoring and enhanced monitoring of IgE levels (total and *Aspergillus* specific IgE), in addition to testing at annual review, even in the absence of clinical symptoms of ABPA could be considered.

Our study showed an association between both *P. aeruginosa* infection and chronic macrolide therapy with subsequent ABPA, which has been shown in an older cross-sectional observational study [6]. Our study also shows that lower lung function, a key indicator of worse respiratory disease, is associated with the subsequent development of ABPA, which has also been shown in a retrospective case–control study (n = 219 cases and controls) [7] and a cross-sectional observational study [6]. It is likely that more severe lung disease with recurrent *P. aeruginosa* and need for chronic macrolide therapy, provides an environment which facilitates *Aspergillus* colonisation and persistence, leading to the development of ABPA. The impact of chronic macrolide therapy itself also needs to be considered, with longitudinal observational data (n = 85) showing an independent association of chronic macrolide use and *Aspergillus* colonisation in CF [8]. Experimental studies have shown that wild type mice treated with antibiotics, developed an allergic respiratory response following *Aspergillus* exposure, which did not happen in mice not treated with antibiotics [23]. These data suggest that patients with lower lung function and recurrent *P. aeruginosa* requiring chronic macrolide therapy might benefit from enhanced surveillance for early diagnosis of ABPA.

Our study showed an association of increased age and reduced risk of developing ABPA. Over the duration of the study, the UK CF Registry reported that the overall prevalence of ABPA in all ages reduced from 8.3% to 7.5%, with the greatest reduction in <16-year-olds, a reduction from 6.9% in 2009 to 3.5% in 2019 [24,25]. The multifaceted improved treatment of CF lung disease, including the introduction of CFTR modulator therapy, is likely to explain the overall reduction in ABPA over the duration of the study.

Our study did not show an association between percentile BMI and subsequent development of ABPA in our longitudinal analysis, whereas an association with lower BMI was shown in another small longitudinal observational study [8]. The results of our study suggest that low BMI is not a risk factor for ABPA. Of note in our baseline cohort analysis, ABPA was associated with a higher BMI, which we hypothesise is most likely due to weight gain from oral steroid treatment, the standard first-line treatment for ABPA [1,21].

In our study population, 48 patients were receiving ivacaftor in 2013, which increased to 79 patients by 2019. Ivacaftor monotherapy was first approved in July 2012 in the UK for people with CF ≥ 6 years old with the G551D gating mutation and by 2019 approval had extended to people with CF with other specified gating and residual function mutations, from the age of 1 year. As only patients with certain *CFTR* genotypes are eligible for ivacaftor, this may be a confounding factor. We aimed to minimise this impact by adjusting our longitudinal complementary log–log model for baseline *CFTR* genotype. In our study, the proportion of children with CF who had a single G551D mutation was 5.8%. The relationship between CF genotype and ABPA in CF is not known, and data are conflicting as to whether pwCF with at least one G551D mutation have less severe phenotype than homozygous F508del patients [26,27,28]. A large UK CFFPR study (n = 11,417), using propensity score matching on baseline clinical characteristics, in the pre-CFTR modulator era, showed no difference in lung function decline between pwCF with at least one G551D mutation compared to pwCF homozygous F508del [29]. In non-CF studies, e.g., in patients with asthma, *CFTR* variants were associated with ABPA [30,31]. In our study, there was no significant difference between *CFTR* genotypes between patients who had, or did not have, ABPA at baseline.

Our observation that ivacaftor is associated with a reduced risk of developing APBA is supported by clinical and laboratory studies. Several studies with up to 3 years of follow-up have shown that ivacaftor is associated with reduced *Aspergillus* colonisation [16,17,32]. The correction of the CFTR protein defect in epithelial cells restores the epithelial membrane ion imbalance, normalising the airway surface liquid creating thinner mucous and improving mucociliary clearance of pathogens including *Aspergillus* [33]. With less colonisation of the airways by *Aspergillus*, the inflammatory response to *Aspergillus* is reduced, reducing the likelihood of ABPA [33]. In a small retrospective study of CF patients treated with ivacaftor (n = 40) for 6 months, a significant reduction in total IgE levels was observed, though this was not sustained [34]. In this small study, there was no significant changes in *Aspergillus* specific IgE or IgG with ivacaftor treatment. There is also a direct effect of restoring the CFTR protein defect in innate immune cells. An ex vivo study by Warris et al. [35] showed that CF phagocytes pretreated with ivacaftor or lumacaftor/ivacaftor had significantly reduced *Aspergillus*-induced inflammation compared to those that were not pretreated. The data suggest that the restoration of the host immune response by CFTR modulators contribute to reduced inflammation and potentially decreases the risk to develop ABPA. Additionally, improved antifungal killing by alveolar macrophages upon treatment with ivacaftor and lumacaftor/ivacaftor in vitro, may be another explanation for reduced *Aspergillus* colonisation [36].

Our study did not show a significant protective association between lumacaftor/ivacaftor and ABPA likely due to the too small numbers to show an effect, as the number of patient-years in our dataset for lumacaftor/ivacaftor were 122, whereas, as for ivacaftor, 427 patient-years were reported.

The effect of the triple combination therapy, elexacaftor–tezacaftor–ivacaftor (ETI) on ABPA is as yet unknown, as the phase 3 clinical trials were not powered to assess the effect on ABPA [18,19,37]. Clinical observations since 2015 show that the prevalence of ABPA in CF is declining in the UK [5], and Europe [38], which parallels the expanded roll out of CFTR modulators. The interim results of the US CF Foundation Patient Registry (CFFPR) real-world study of the impact of ETI showed a significant reduction in the prevalence of *Aspergillus* positive respiratory samples from 18.7% to 4% one year after starting treatment, as well as a reduction in pulmonary exacerbations and bacterial infections [19]. Two single-centre retrospective studies showed a reduction in total IgE (but not *Aspergillus* specific IgE or absolute eosinophil counts) in CF patients 12 months after starting ETI [39,40], as well as a rapidly reduced ratio of *Aspergillus* positive sputum cultures [40]. An ex vivo study of 21 patients with CF showed that ETI was able to reduce the aberrant inflammation induced by *Aspergillus*, and bacterial pathogens, as well as to reduce total serum IgE [41]. No real-world data studies have yet been published on the impact of ETI on ABPA, but studies are ongoing including large-scale real-world studies assessing the impact of ETI in the UK, the RECOVER trial [20], and the US CFFPR trial [19].

Key drawbacks of our study are those of all observational studies in that while measures were taken to limit the impact of confounding with the statistical models used, not all measured or unmeasured confounding factors can be accounted for. In this study, certain data were not available such as, type of respiratory sample, number of positive samples in a given year, and species of *Aspergillus*. Blood parameters of atopy were not available such as total IgE, *Aspergillus*-specific IgE, or eosinophilia. Other risk factors that this study was not designed to evaluate, but are nevertheless important, are other genetic risk factors other than CFTR genotype, immune response genes [42,43], and environmental risk factors, as high *Aspergillus* exposure in the home environment has been shown to be associated with ABPA [44].

## 5. Conclusions

Whilst considering the limitations of our observational research, we have identified key risk factors for ABPA in children with CF, including *Aspergillus* colonisation, lower lung function, and *P. aeruginosa* infection. These risk factors may help to design more targeted monitoring to allow for the early diagnosis and treatment of ABPA. This is the first study to show a protective association of ivacaftor and development of ABPA. Real-world longitudinal cohort studies are needed to assess the impact of the now widely used CFTR modulator treatment (ETI) on the incidence of ABPA, as well as on the duration of this protective effect.

## Figures and Tables

**Table 1:** Baseline characteristics of 1612 patients with CF aged 8 to 17 years in 2009.

	Whole Cohort	ABPA Absent	ABPA Present	*p*-Value
	(n = 1612)	(n = 1420)	(n = 192)	
**Clinical characteristics (2009)**				
**Male sex**	829	729	100	0.85
**Number (%)**	(51.4%)	(51.3%)	(52.1%)	
**Age in years**	11.9	11.9	12.4	0.008
Mean (SD)	(2.51)	(2.51)	(2.50)	
***CFTR*** genotype (n = 1601) *				0.32
F508del/F508del	895	786	109	
Number, (%)	55.9%	55.8%	56.8%	
F508del/Other	593	528	65	
Number, (%)	37.0%	37.5%	33.9%	
Other	113	95	18	
Number, (%)	7.1%	6.7%	9.4%	
**ppFEV_1_** (n = 1444) *	80.3	80.9	76.0	0.0003
Mean (SD) (range)	(16.9) (18.5–135.7)	(16.9) (18.5–135.7)	(16.4) (30.9–113.1)	
**pBMI** (n = 1550) *	49.4	48.8	53.6	0.04
Mean (SD) (range)	(29.0) (0–99.9)	(29.1) (0–99.9)	27.9 (0.9–99.3)	
**Respiratory disease severity markers (2009)**				
**Oxygen therapy** (n = 1469) *	44	30	14	<0.0001
Number, (%)	(3.0%)	(2.3%)	(7.9%)	
**Non-invasive ventilation**	27	16	11	<0.0001
(n = 1445) * Number, (%)	(1.9%)	(1.3%)	(6.2%)	

ppFEV_1_ = percentage predicted forced expiratory volume in 1 s. pBMI = percentile body mass index. Categorical variables: *p*-value calculated with Chi-squared test. Continuous variables: *p*-value calculated with independent sample *t*-test. * Number in analysis, given if number less than total in group (i.e., if any missing data).

**Table 2:** Baseline (2009) cross-sectional analysis with multivariable logistic regression model adjusted for baseline age, gender, and genotype.

Factors Associated with ABPA in 2009: (n = 1356) *		
**Clinical characteristics** OR (95% CI)		
**ppFEV_1_**	0.998 (0.986–1.009)	*p* = 0.65
**pBMI**	1.009 (1.003–1.015)	*p* = 0.004
**Infection** OR (95% CI)		
*Pseudomonas aeruginosa* **	0.92 (0.62–1.38)	*p* = 0.70
***Aspergillus* spp.** **	2.00 (1.15–3.49)	*p* = 0.01
**Treatments** OR (95% CI)		
**IV antibiotic requirement** ***	3.59 (2.41–5.36)	*p* < 0.0001
**Chronic oral macrolide**	1.24 (0.87–1.76)	*p* = 0.24
**Pancreatic enzyme supplementation**	1.97 (0.81–4.78)	*p* = 0.13

* Number in analysis (256 patients with ≥1 missing variable; therefore, these patients were excluded from this analysis). ppFEV_1_ = percentage predicted forced expiratory volume in 1 s. pBMI = percentile body mass index. ** ≥1 positive respiratory sample/12 months. *** yes/no requirement for antibiotics.

**Table 3:** Clinical predictors for ABPA. Longitudinal analysis (2009–2019) of patients without ABPA in 2009 to determine risk factors for subsequent development of ABPA using a complementary log-log regression model.

ABPA Predictors: (n = 1394) *		
Patient Characteristics: OR (95% CI)		
**Age**	0.89 (0.87–0.91)	*p* < 0.0001
***CFTR* genotype** F508del heterozygous (vs. F508del homozygous) Other (vs. F508del homozygous)	1.07 (0.93–1.22) 0.82 (0.62–1.07)	*p* = 0.16
**Male gender**	1.45 (1.28–1.65)	*p* < 0.0001
**Disease characteristics at annual review preceding year of ABPA diagnosis:** OR (95% CI)		
**ppFEV_1_**	0.98 (0.98–0.99)	*p* < 0.0001
**pBMI**	1.000 (0.999–1.001)	*p* = 0.66
**Infection at annual review preceding year of ABPA diagnosis:** OR (95% CI)		
***Pseudomonas aeruginosa*** **	1.31 (1.15–1.49)	*p* < 0.0001
***Aspergillus* colonisation** **	1.47(1.28–1.69)	*p* < 0.0001

* Number in analysis (26 patients excluded due to missing data (11 missing *CFTR* genotype, 13 missing ppFEV_1_ every year, 1 missing pBMI every year, and 1 missing other variable every year). OR = Odds Ratio, CI = confidence interval. ** ≥1 positive respiratory sample/12 months.

**Table 4:** Treatments predictors for ABPA. Longitudinal analysis (2009–2019) of patients without ABPA in 2009 using a complementary log–log regression model.

ABPA Predictors: (n = 1394) *		
Treatments at Annual Review Preceding Year of ABPA Diagnosis: OR (95% CI)		
Ivacaftor (Kalydeco^®^)	0.46 (0.26–0.80)	*p* = 0.006
Lumacaftor/ivacaftor (Orkambi^®^)	0.64 (0.29–1.44)	*p* = 0.28
Pancreatic enzyme supplements	0.86 (0.68–1.09)	*p* = 0.22
Total IV antibiotic days	1.000 (0.996–1.000)	*p* = 0.24
Chronic oral macrolide use	1.30 (1.15–1.48)	*p* < 0.0001

* Number in analysis (26 patients excluded due to missing data (11 missing *CFTR* genotype, 13 missing ppFEV_1_ every year, 1 missing pBMI every year, and 1 missing other variable every year). OR = Odds Ratio, CI = confidence interval, IV = intravenous.

## Data Availability

UK CF Registry Data are available using a formal review process and the relevant information can be found here UK CF Registry Apply for Data.

## Supplementary Materials

The following supporting information can be downloaded at: https://www.mdpi.com/article/10.3390/jof11020116/s1. Supplementary File S1: Full list of variables as provided by the UK CF Registry; Table S1: Numbers of patients started on CFTR modulator therapy in the longitudinal analyses (those who were ABPA negative in 2009).
