# Supplementary material for: Longitudinal Study on Clinical Predictors for Allergic Bronchopulmonary Aspergillosis in Children and Young People with Cystic Fibrosis Highlights the Impact of Infection with Aspergillus and Pseudomonas and Ivacaftor Treatment
Source: J Fungi (Basel). 2025 Feb 4;11(2):116. doi: 10.3390/jof11020116 (PMC11855986; doi:10.3390/jof11020116)
Supplement: Supplementary file 1 [file jof-11-00116-s001.zip › jof-3371519-supplementary.pdf]

Supplemental file

Supplementary File S1: Full list of variables as provided by the UK CF Registry

Fully anonymised data from the CF Registry was requested for the years 2009 to 2019 (01.01.09-31.12.19) from all participants aged 8 to 17 years in the years 2009-2010. Variables:

- Age at annual review encounter
- Diagnosis- age at diagnosis and presence of respiratory infection (acute or persistent)
- Gender
- Ethnicity
- CF gene mutation class
- BMI percentile (%)
- Baseline lung function as measured by $ppFEV_1$, and $ppFEV_1$ in subsequent years
- Total number of respiratory samples since last annual review (number in each category of sputum, cough/throat/nose, and bronchoscopy samples)
- *Aspergillus fumigatus* positive respiratory samples since last annual review Y/N and *Aspergillus* species positive respiratory samples since last annual review Y/N
- *Pseudomonas aeruginosa* (Pa) positive respiratory swabs that year Y/N
    - Mucoid/ non-mucoid
- Intermittent/ chronic Pa
- Other bacterial co-infections that year:
    - *Staphylococus aureus* Y/N (Chronic/intermittent)
    - *Burkholderia cepacia* complex Y/N
    - Non-*tuberculous mycobacteria* Y/N
    - Other Y/N
- Number of hospitalisations for intravenous therapy (IV admissions)
- Number of intensive care unit admissions
- Number of home intravenous courses (home IV courses)
- Number of hospitalisations for non-intravenous therapy (non-IV admissions)
- Allergic bronchopulmonary aspergillosis (ABPA) Y/N
- Chronic treatment with antibiotics Y/N
    - Inhaled anti-pseudomonal antibiotics
    - Oral flucloxacillin
    - Oral anti-pseudomonal antibiotics
- Chronic treatment with steroids Y/N
- CFTR modulator therapy Y/N and which type: ivacaftor alone/lumacaftor- ivacaftor/ tezecaftor-ivacaftor/elexacaftor-tezacaftor-ivacaftor

- Oxygen therapy since last annual review: continuous, or nocturnal +/- with exertion, or during exacerbation or PRN
- Non-invasive ventilation therapy in the last year Y/N
- Presence of complications:
    - CF related diabetes (CFRD) Y/N
    - Pancreatic insufficiency Y/N
- Evaluation for lung transplantation in that year (accepted/declined/deferred) Y/N
- Lung transplant in that year Y/N
- Death Y/N
- Primary cause of death

Supplementary Table S1: Numbers of patients started on CFTR modulator therapy in the longitudinal analyses (those who were ABPA negative in 2009)

Year	Number of patients on ivacaftor	Number of patients on lumacaftor/ ivacaftor	Number of patients on tezacaftor/ ivacaftor	Number of patients on CFTR modulator (any)
**2018**	79	72	0	151
**2017**	77	35	1	113
**2016**	77	15	0	92
**2015**	74	0	0	74
**2014**	72	0	0	72
**2013**	48	0	0	48
**2012**	0	0	0	0

Total number of patients in analysis=1394. 2012 to 2018 shown as 2018 last year when CFTR modulator data included in analysis. No patients were on CFTR modulator therapy prior to 2013.
